# Arthritis Accompanying Ophthalmia Neonatorum

**Published:** 1902-06-07

**Authors:** 


### ARTHRITIS ACCOMPANYING OPHTHALMIA NEONATORUM.

At the last meeting of the Society for the Study of Disease in Children, Dr. C. O. Hawthorne related a case of arthritis accompanying ophthalmia neonatorum, and referred to other instances in which this conjunction had been observed. It seems from the account given that the father of the patient had gonorrhœa two months before the child was born, that the mother was subsequently affected, and that the child began to suffer from purulent ophthalmia when two days old; so the nature of the primary disease is hardly open to doubt. When the infant was a fortnight old the right shoulder joint became considerably swollen, and the right wrist slightly so. This did not appear to cause much discomfort, and the swelling subsided in the course of a fortnight. When six weeks old the child was seen by Dr. Hawthorne. There was then a slight muco-purulent discharge from the conjunctivæ, and the right cornea showed a small abrasion from which a diffuse haziness spread through the corneal substance. The joints first attacked had regained their normal condition, but now the left elbow joint was considerably enlarged, the swelling extending down the radial side of the forearm for a considerable distance. The infant made a rapid recovery under local treatment to the eye and fixation of the limb. Dr. Hawthorne suggests that the readiness with which these cases recover under treatment as compared with the obstinate and prolonged evil effects which frequently attend the usual form of gonorrhœal urethritis, is due to the ease with which a purulent ophthalmia can be successfully treated as compared with the difficulty of bringing to a close the opportunities for absorption of purulent discharge offered by the urethra and its related glandular recesses. If this be so it should emphasise the importance of checking the urethral discharge in all cases of gonorrhœal arthritis.

